# Evaluating the Quality and Features of Visual Acuity Apps Using the Mobile App Rating Scale: Systematic Review

**DOI:** 10.2196/65997

**Published:** 2025-12-12

**Authors:** P Connor Lentz, Emily Dorairaj, Pranav Vasu, Isabella Wagner, Jaxson Jeffery, Farha Deceus, Nithya Boopathiraj, Yazan Abubaker, Darby D Miller, Antonio Jorge Forte, Syril Dorairaj

**Affiliations:** 1 New York Eye and Ear Infirmary of Mount Sinai, New York, NY, United States; 2 FAU Charles E. Schmidt College of Medicine, Boca Raton, FL, United States; 3 Creighton University School of Medicine, Phoenix, AZ, United States; 4 University of Florida College of Medicine, Gainesville, FL, United States; 5 Mayo Clinic Alix School of Medicine, Scottsdale, AZ, United States; 6 Department of Ophthalmology, Mayo Clinic Florida, 4500 San Pablo Road South, Jacksonville, FL, 32224, United States, 1 (904) 953-2000; 7 Bascom Palmer Eye Institute, University of Miami, Miami, FL, United States; 8 Department of Plastic Surgery, Mayo Clinic Florida, Jacksonville, FL, United States

**Keywords:** visual acuity, ophthalmology, telehealth, teleophthalmology, app, mobile app, mhealth, rating scale, eye care, patient, clinical, Mobile App Rating Scale, MARS, clinical setting, eye, telemedicine, technology, technology-based, eye health, mobile health

## Abstract

**Background:**

Mobile visual acuity (VA) apps have emerged as valuable tools in both clinical and home settings, particularly in the context of expanding teleophthalmology. Despite the growing number of apps available to measure visual acuity, studies evaluating their overall quality, functionality, and clinical relevance are limited.

**Objective:**

This study aimed to systematically evaluate the quality and features of mobile VA apps available on iOS and Android platforms using the clinically validated Mobile App Rating Scale (MARS).

**Methods:**

A comprehensive search of the Google Play Store and Apple App Store was conducted between January 2024 and March 2024 using standardized search terms. Eligible apps included free, English-language VA testing tools not requiring external devices. App characteristics and features were extracted. Each app was independently evaluated by 2 trained reviewers using MARS, which rates engagement, functionality, aesthetics, information quality, and subjective quality on a 5-point scale.

**Results:**

Of the 725 apps initially identified, 44 met the inclusion criteria, with 23 from the Google Play Store and 21 from the Apple App Store. The most common VA test optotypes used were Tumbling E (n=21; 48%), Snellen Chart (18/44; 41%), and Landolt C (n=14; 32%). Common supplemental features included color vision testing (n=20; 46%), astigmatism tests (n=13; 30%), Amsler grid (n=13; 30%), and contrast testing (n=12; 28%). The average MARS scores were comparable across platforms: 3.04 (SD 0.80) for Android and 3.02 (SD 0.84) for iOS. Functionality received the highest ratings (mean 3.65, SD 0.75 for Android; mean 3.71, SD 0.82 for iOS), while subjective quality received the lowest (mean 2.09, SD 1.01 for Android; mean 2.21, SD 1.01 for iOS). Few apps had undergone clinical validation. Only Apple App Store apps demonstrated significant correlations between MARS scores and app store star ratings.

**Conclusions:**

VA apps exhibited considerable heterogeneity in quality, functionality, and clinical use. Total mean MARS scores were similar between the Google Play Store and the Apple App Store, suggesting that neither platform consistently offers superior app quality. While many apps are technically sound, low subjective-quality scores and a lack of clinical validation limit their current use in professional practice. These findings underscore the need for more rigorous app development and validation standards to improve their relevance and reliability in teleophthalmology.

## Introduction

In ophthalmology, visual acuity (VA) tests serve as vital clinical tools for both monitoring general visual health and the diagnosis of visual impairment. While traditionally performed in a clinical setting by an eye-care provider, in recent years, there has been a large-scale growth of technology-based clinical assessments [1]. This emphasis on expanding teleophthalmology serves to overcome barriers experienced by many visually impaired patients, which may include limited access to eye care providers or an inability to afford regular eye exams due to insurance constraints [2].

VA mobile apps are 1 form of teleophthalmology that has great potential to be used by both providers and patients to monitor eye health [3]. These apps have use in the hospital setting as they are easily accessible to ophthalmologists and nonophthalmologist providers to quickly assess changes in VA. They can also be downloaded by patients to be used at home and provide educational information and instructions on when it is appropriate to have a formal eye exam. The inclusion of other visual tests, such as those for color vision, astigmatism, macular degeneration, and others, greatly enhances the benefit of these apps for patient and provider use.

The influx of numerous visual acuity apps has prompted recent literature evaluating their usefulness and validity. Steren et al [1] assessed the accuracy of 6 VA apps available on both iPad and iPhone; however, none of the apps were deemed functional for telehealth use. Kawamoto et al [4] reviewed 24 apps from both the Google Play Store and the App Store, identifying only 3 apps they considered appropriate for clinical use. These publications have called into question the quality and efficacy of currently available VA apps.

The Mobile App Rating Scale (MARS) was created as a systematic method to evaluate and ensure high quality of health-related apps. The MARS rates apps through 5 categories: engagement, functionality, aesthetics, information, and subjective quality and has been found to have high inter-rater reliability [5]. It is the most frequently used scale to rate health apps in research [6] and has previously been used to evaluate apps related to hematologic diseases, food allergies, smoking cessation, depression, pregnancy, and many more [7-12].

This review aims to use the MARS to comprehensively analyze existing VA apps. By identifying key areas where existing apps must be improved, this review may serve as a guideline for future VA app development.

## Methods

### Search Strategy

A systematic search was performed on the Google Play Store (Android) and the Apple App Store (iOS) by 2 authors on Android (version 11) and iPhone (iOS version 17.5.1) mobile devices beginning January 1, 2024, to March 31, 2024. Search terms included “Visual Acuity,” “Eye Exam,” “Eye Test,” and “Vision Test.” All search results were manually recorded and duplicates within each app store were removed. This systematic review was conducted in accordance with PRISMA (Preferred Reporting Items for Systematic Reviews and Meta-Analyses) guidelines (Checklist 1).

### App Selection

Identical apps available on both the Google Play Store and the Apple App Store were considered separate for screening, rating, and analysis due to potential differences in demographics and app characteristics between systems. Initial screening excluded apps unrelated to visual acuity testing and non-English apps. The unrelated apps consisted primarily of color vision tests, eye exercises, eye health monitoring, ophthalmology calculators, eye photography, blue light filters, assistance for low-vision individuals, and children’s games. To ensure accessibility for general patients and providers, paid apps and those requiring external devices were also excluded.

The remaining apps were downloaded and evaluated for eligibility. Apps that were nonfunctional or inaccessible after download were excluded, as were apps lacking a VA testing function. Apps that met all inclusion criteria were included for data extraction and MARS evaluation.

### Data Extraction

Data extraction was performed by 2 medical students, with one assigned to each app store, using the US version of each app store. General characteristics of each eligible app were collected from their respective app stores. The features described in the app store descriptions were verified through download and testing. The date of the most recent update was recorded for all apps on the same day to ensure accurate analysis of update history. Characteristics of visual acuity tests, other eye examinations, and additional app features were also documented. Features requiring in-app purchases were excluded from the app characteristic results.

### MARS Assessment

Prior to performing app quality assessment, all raters viewed the MARS training video, which provided standardized instruction on rating and classifying apps [13]. Two raters evaluated Google Play Store apps on Android smartphones, and 2 separate raters rated Apple Store apps on iPhones. All raters were medical students based in the United States and evaluated apps downloaded from the US versions of the respective app stores. Each app was downloaded and tested for at least 15 minutes by each rater. Upon completion of testing, the MARS was applied. Ratings for each app were initially conducted independently and then compared for agreement between the 2 raters. In the case of disagreement, resolution was reached through the intermediation of a third rater.

The MARS [5] consists of 23 items across five sections:

1. Engagement: Is the app fun, interesting, customizable, interactive, and well-targeted to its audience?
2. Functionality: Is the app functional, easy to learn, with logical flow and intuitive design?
3. Aesthetics: How are the graphic design, overall visual appeal, color scheme, and stylistic consistency?
4. Information: Does the app contain high-quality information from a credible source?
5. App subjective quality: What personal rating would the rater give this app?

The raters scored each item on a scale of 1=poor to 5=excellent. One item in the “Information” section evaluates the credibility of the app developer; for this, each rater conducted a web-based search of the developer’s name to verify the app’s source. To answer another item, the app name was entered into a MEDLINE search to identify any associated clinical trials. The MARS also includes a final app-specific section regarding raters’ opinions on how the app may influence attitudes toward a target health behavior. This section was not performed for this study due to its low applicability to provider-targeted apps.

### Statistical Analysis

For each app, the mean MARS score and SD were calculated for each section, along with a total mean score encompassing all 5 sections. To assess differences in app quality between the app stores, 2-tailed *t* tests were performed. Pearson correlation coefficients were used to examine the associations between mean scores for each MARS section, the total number of reviews, and the average star rating. These correlations were calculated separately for the Google Play Store and the Apple App Store.

### Ethical Considerations

This review did not involve human subjects or identifiable data; therefore, institutional review board approval was not required.

## Results

### App Selection

**Figure 1.**
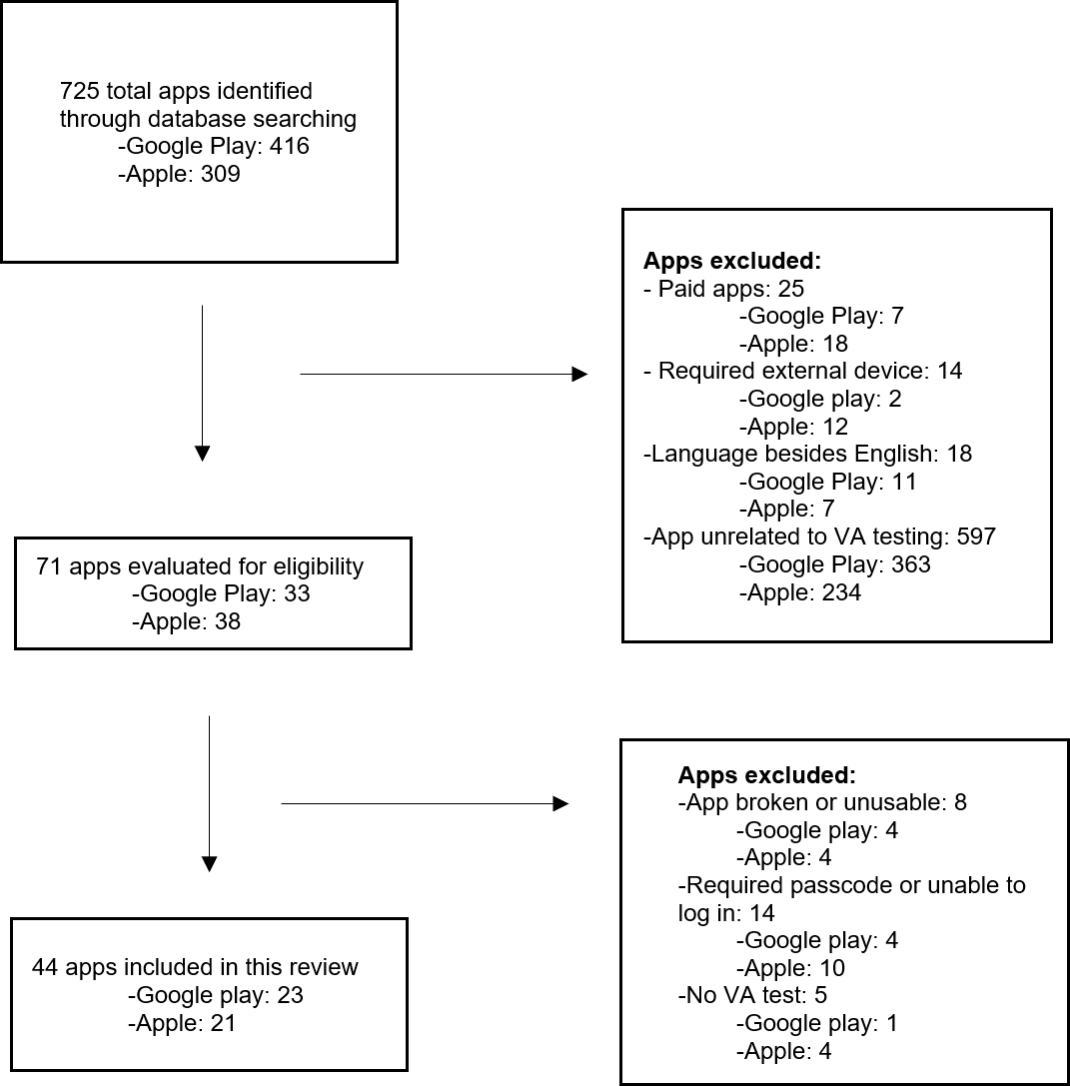
Summary of app selection process. VA: visual acuity.

The results of the app selection process are shown in Figure 1. A total of 725 apps were identified in the initial search of the Apple App Store and Google Play Store. The initial screening process resulted in 71 apps that were tested for eligibility. After applying the final exclusion criteria, 44 apps were included in the study: 23 (52%) from the Google Play Store and 21 (48%) from the Apple App Store. Five of these apps were available on both app stores: Asia Retina (Skubbs) [14], Eye Handbook (Cloud Nine Development) [15], Eye Patient (Cloud Nine Development) [16], Smart Optometry–Eye Tests (Smart Optometry doo) [17], and WHOeyes (World Health Organization) [18].

### App Characteristics and Features

App characteristics are featured in Table 1. From the 44 apps, 29 (66%) across both app stores were targeted for use by eye care providers. The Apple App Store had a higher proportion of apps (17/21; 81%) created over 3 years ago compared to the Google Play Store (9/23 apps; 39%). Over half of the Apple apps (13/21; 52%) had not been updated in the last 2 years, whereas the largest proportion of Android apps (9/23; 39%) had been updated within the past 6 months to 1 year. Regarding language support, Spanish was the most common additional language, featured in 7 of 44 (16%) apps across both stores. However, Japanese was the most frequently featured other language among Apple apps (4/21; 19%) due to the presence of several Apple VA app publishers from Japan. Over half of Android apps (12/23; 52%) had advertisements within the app and 4 of 23 (17%) apps had features that required payment to access. Eight of 21 (38%) apps in the Apple Store had advertisements within the app and only 1 of 21 (5%) apps had paid features.

**Table 1.** General characteristics of evaluated visual acuity (VA) apps by platform (N=44).

App characteristic	Google Play Store (n=23), n (%)	Apple App Store (n=21), n (%)	Total, n (%)
Target audience			
Providers	14 (61)	15 (71)	29 (66)
Patients	6 (26)	3 (14)	9 (21)
Both	3 (13)	3 (14)	6 (14)
App release date (y)			
<1 year	5 (22)	1 (5)	6 (14)
1‐2 years	6 (26)	0 (0)	6 (14)
2‐3 years	3 (13)	1 (5)	4 (9)
>3 years	9 (39)	17 (81)	26 (59)
Unknown	0 (0)	2 (10)	2 (5)
Most recent update			
<1 month	3 (13)	1 (5)	4 (9)
1‐3 months	2 (9)	2 (10)	4 (9)
3‐6 months	4 (17)	1 (5)	5 (11)
6 months to 1 year	9 (39)	2 (10)	11 (25)
1‐2 years	5 (22)	2 (10)	7 (16)
>2 years	0 (0)	11 (52)	11 (25)
Unknown	0 (0)	2 (10)	2 (5)
Additional language options			
Spanish	4 (17)	3 (14)	7 (16)
Portuguese	2 (9)	1 (5)	3 (7)
Japanese	1 (4)	4 (19)	5 (11)
Russian	3 (13)	2 (10)	5 (11)
Chinese	2 (9)	3 (14)	5 (11)
Arabic	1 (4)	2 (10)	3 (7)
French	2 (9)	2 (10)	4 (9)
Other	2 (9)	2 (10)	4 (9)
Advertisements within the app	12 (52)	8 (38)	20 (46)
Paid features within the app	4 (17)	1 (5)	5 (11)

A list of tests and other features of the VA apps is shown in Table 2. The Tumbling E was the most common optotype (21/44; 48%) of the VA tests featured among all apps, followed by Snellen Chart (18/44; 41%) and Landolt C (14/44 apps; 32%). The total percent of all VA tests is greater than 100% due to many apps featuring multiple optotypes. “Other” optotypes of visual acuity tests consisted of primarily words, sentences, and numbers. The most common feature outside of VA tests was color-vision tests, typically in the form of Ishihara color plates, which were included in 20 (46%) of the total VA apps. Examples of “other tests” included visual field tests, duochrome tests, and Schober tests.

**Table 2.** Tests and features of the visual acuity (VA) apps by platform (N=44).

App test or feature	Google Play Store (n=23), n (%)	Apple App Store (n=21), n (%)	Total, n (%)
Snellen Chart	10 (44)	8 (38)	18 (41)
ETDRS	0 (0)	1 (5)	1 (2)
Tumbling E	10 (44)	11 (52)	21 (48)
Landolt C	8 (35)	6 (29)	14 (32)
Pediatric or pictures	3 (13)	5 (24)	8 (18)
Other	7 (30)	6 (29)	13 (30)
Color vision	11 (48)	9 (43)	20 (46)
Contrast	7 (30)	5 (24)	12 (27)
Astigmatism (dial or line test)	9 (39)	4 (19)	13 (30)
Amsler grid	6 (26)	7 (33)	13 (30)
Pupillary distance	1 (4)	1 (5)	2 (5)
Optokinetic drum	4 (17)	2 (10)	6 (14)
Worth 4 Dot	4 (17)	2 (10)	6 (14)
Educational materials	7 (30)	5 (24)	12 (27)
Calculators	2 (9)	1 (5)	3 (7)
Other tests	8 (35)	8 (38)	16 (36)

### App Quality Ratings

The MARS scores for each app are reported in Tables3  4. On a 1-to-5 scale, the overall mean (SD) rating across all apps on the Google Play Store was found to be 3.04 (0.80; 23/44, 52%). In comparison, apps on the Apple App Store had an overall mean (SD) rating of 3.02 (0.84; 21/44, 48%). No statistically significant difference was found between the total mean MARS scores of apps from each app store.

**Table 3.** Average Mobile App Rating Scale (MARS) scores for Google Play Store visual acuity apps.

App name (developer)	Engagement, mean (SD)	Functionality, mean (SD)	Aesthetics, mean (SD)	Information, mean (SD)	Subjective quality, mean (SD)	Total, mean (SD)
Asia Retina (Skubbs) [14]	3.00 (0.71)	4.25 (0.50)	4.33 (0.58)	3.80 (0.45)	2.00 (0.82)	3.48 (0.98)
Easy Vision Exam (Barbaroi Ware) [19]	3.40 (0.55)	4.50 (0.58)	3.67 (0.58)	3.67 (2.31)	1.50 (1.00)	3.35 (1.11)
Eye chart mobile (DcRovshan) [20]	1.20 (0.45)	2.25 (1.26)	1.33 (0.58)	1.75 (0.96)	1.00 (0.00)	1.51 (0.50)
Eye exam (Andrei Brusentsov) [21]	3.00 (0.71)	4.00 (0.00)	3.00 (1.00)	3.50 (1.00)	1.50 (0.58)	3.00 (0.94)
Eye Handbook (Cloud Nine Development) [15]	4.60 (0.55)	4.75 (0.50)	4.00 (0.00)	4.86 (0.38)	5.00 (0.00)	4.64 (0.39)
Eye Health Manager (Nikita Tyart) [22]	3.40 (0.55)	4.25 (0.50)	3.67 (0.58)	3.50 (1.73)	2.75 (0.96)	3.51 (0.54)
Eye Patient (Cloud Nine Development) [16]	4.40 (0.55)	4.50 (0.58)	4.33 (0.58)	4.67 (0.52)	3.50 (1.00)	4.28 (0.45)
Eye Test–Visual Optometry (Toprank) [23]	3.60 (0.89)	3.50 (0.58)	3.67 (0.58)	3.00 (1.41)	1.75 (0.50)	3.10 (0.80)
Eye Test Exam (M’n Labs) [24]	1.20 (0.45)	3.25 (0.96)	1.67 (0.58)	1.67 (0.58)	1.00 (0.00)	1.76 (0.88)
Eye Test Girls (fmroid) [25]	2.60 (0.55)	3.75 (0.50)	2.33 (0.58)	2.50 (1.29)	1.00 (0.00)	2.44 (0.98)
EyeCharts–Visual Acuity (JUAN CLC) [26]	2.60 (0.55)	2.25 (0.50)	1.67 (0.58)	2.50 (0.84)	1.75 (0.50)	2.15 (0.43)
EyeD–Smart Blink Reminder (Autoyos) [27]	3.40 (0.55)	4.00 (0.00)	3.67 (0.58)	3.50 (0.84)	2.25 (0.96)	3.36 (0.66)
Eyesight: Eye Exercise & Test (Arka iTech) [28]	3.60 (0.55)	3.75 (0.50)	3.00 (0.00)	3.00 (0.82)	1.75 (0.50)	3.02 (0.79)
Optical visual test (Alejo Apps) [29]	1.80 (0.45)	3.25 (0.50)	2.67 (0.58)	2.00 (1.00)	1.00 (0.00)	2.14 (0.86)
OptoCharts—all eye tests (Shadi AboelNaga) [30]	2.40 (0.55)	2.25 (0.50)	2.00 (0.00)	2.33 (1.15)	1.50 (0.58)	2.10 (0.37)
Optometry eye distance measure (ds optometry) [31]	2.40 (0.55)	3.00 (0.00)	3.00 (0.00)	3.25 (1.50)	1.50 (0.58)	2.63 (0.70)
Peek Acuity (Peek Vision) [32]	3.60 (0.55)	4.50 (0.58)	4.67 (0.58)	4.25 (0.96)	4.00 (0.00)	4.20 (0.42)
Smart Optometry–Eye Tests (Smart Optometry doo) [17]	3.40 (0.55)	4.00 (0.00)	4.33 (0.58)	4.00 (0.71)	3.00 (0.00)	3.75 (0.54)
Snellen Chart (Joao Meneses) [33]	2.40 (1.14)	4.00 (0.00)	3.00 (0.00)	3.67 (2.31)	2.25 (0.96)	3.06 (0.77)
Snellen Chart (Fonlow) [34]	2.60 (0.55)	3.75 (0.50)	2.67 (0.58)	3.33 (2.08)	2.25 (0.96)	2.92 (0.61)
Vision Tests (Appzileiro) [35]	2.80 (0.45)	3.00 (0.82)	3.33 (0.58)	2.60 (1.14)	1.75 (0.50)	2.70 (0.59)
VisionCare (VisionCare Team) [36]	3.60 (0.55)	3.00 (0.82)	4.67 (0.58)	3.00 (1.00)	1.50 (0.58)	3.15 (1.15)
WHOeyes (World Health Organization) [18]	3.20 (0.84)	4.25 (0.50)	4.33 (0.58)	4.40 (0.55)	2.50 (1.29)	3.74 (0.85)
Overall, mean (SD; range)	2.97 (0.86; 1.2‐4.6)	3.65 (0.75; 2.25‐4.75)	3.26 (1.00; 1.33‐4.67)	3.25 (0.88; 1.67‐4.86)	2.09 (1.01; 1.00‐5.00)	3.04 (0.80; 1.51‐4.64)

**Table 4.** Average Mobile App Rating Scale (MARS) scores for apple app store visual acuity apps.

App name (developer)	Engagement, mean (SD)	Functionality, mean (SD)	sthetics, mean (SD)	Information, mean (SD)	Subjective quality, mean (SD)	Total, mean (SD)
Asia Retina (Skubbs) [14]	2.60 (0.89)	4.00 (0.00)	4.00 (0.00)	3.60 (0.55)	2.50 (0.58)	3.34 (0.74)
Easy Vision Log (Kentaro Hosokawa) [37]	3.40 (0.55)	4.00 (0.00)	3.67 (0.58)	3.60 (0.89)	1.75 (0.50)	3.28 (0.88)
Eye Handbook (Cloud Nine Development) [15]	4.20 (0.84)	4.00 (0.00)	4.00 (0.00)	4.67 (0.52)	4.25 (0.50)	4.22 (0.27)
Eye Patient (Cloud Nine Development) [16]	4.00 (0.71)	4.75 (0.50)	4.33 (0.58)	4.50 (0.84)	4.00 (0.00)	4.32 (0.32)
Eye Test 2020 (Isam Al Saadi) [38]	2.00 (0.71)	3.25 (0.50)	2.33 (0.58)	2.83 (0.75)	1.75 (0.50)	2.43 (0.61)
Eye Test Snellen Ishihara (Claire Holmes) [39]	1.20 (0.45)	3.75 (0.50)	2.33 (0.58)	2.67 (1.15)	1.00 (0.00)	2.19 (1.13)
Eye XM Virtual Visit (Eyenexo LLC) [40]	2.60 (0.89)	2.75 (0.50)	3.00 (0.00)	3.00 (0.00)	1.75 (0.50)	2.62 (0.52)
E-Y-E-Check (yinswork) [41]	2.60 (0.89)	3.75 (0.50)	3.00 (0.00)	3.20 (0.45)	1.75 (0.50)	2.86 (0.75)
eyeCuity: Near Vision Card (Isdin Oke) [42]	1.80 (0.84)	3.75 (0.50)	3.33 (0.58)	3.20 (0.45)	1.75 (0.50)	2.77 (0.93)
Eyes Checker (Isam Al Saadi) [43]	1.60 (0.89)	3.00 (0.00)	1.67 (1.15)	2.40 (0.55)	1.75 (0.50)	2.08 (0.60)
Eye’s Test (Takeshi Kawai) [44]	2.00 (0.71)	4.00 (0.00)	3.33 (0.58)	3.00 (0.00)	1.75 (0.50)	2.82 (0.94)
EyeTesterFree (FUSO PRECISION) [45]	1.20 (0.45)	2.00 (0.82)	2.00 (0.00)	1.67 (0.58)	1.00 (0.00)	1.57 (0.46)
eyeTests Easy (George Kong software) [46]	2.00 (0.71)	2.00 (0.00)	1.67 (0.58)	2.50 (0.58)	1.25 (0.50)	1.88 (0.46)
Home vision check (Zachary Silver) [47]	2.80 (0.84)	4.00 (0.00)	3.33 (0.58)	3.83 (0.41)	2.25 (0.96)	3.24 (0.73)
NYU^a^ Langone Eye Test (NYU Langone Medical Center) [48]	2.80 (0.84)	4.25 (0.50)	4.00 (0.00)	3.87 (0.33)	2.25 (0.96)	3.43 (0.86)
OcularCheck: Acuity Exam (Project ORBIS International Inc) [49]	3.00 (0.71)	4.25 (0.50)	3.67 (0.58)	3.86 (0.69)	2.50 (0.58)	3.45 (0.70)
REST^b^ Rapid Eye Screening Test (ZU QUAN IK) [50]	2.60 (0.55)	4.00 (0.00)	3.67 (0.58)	3.00 (0.00)	1.75 (0.50)	3.00 (0.89)
Smart Optometry–Eye Tests (Smart Optometry) [17]	3.20 (0.45)	4.00 (0.00)	4.00 (0.00)	3.67 (0.52)	2.75 (0.50)	3.52 (0.54)
Telehealth Eye Test (Just for Eyes) [51]	1.40 (0.89)	2.75 (0.50)	2.00 (0.00)	2.40 (0.55)	1.00 (0.00)	1.91 (0.71)
WHOeyes (World Health Organization) [18]	3.20 (0.45)	4.75 (0.50)	4.00 (0.00)	4.00 (0.58)	3.00 (0.82)	3.79 (0.70)
LooC—Mobile eye test (mathHeartCode UG[haftungsbeschraenkt]) [52]	4.40 (0.55)	5.00 (0.00)	5.00 (0.00)	4.17 (1.17)	4.75 (0.50)	4.66 (0.37)
Overall, mean (SD; range)	2.60 (0.93; 1.20‐4.40)	3.71 (0.82; 2.00‐5.00)	3.25 (0.94; 1.67‐5.00)	3.32 (0.76; 1.67‐4.67)	2.21 (1.05; 1.00‐4.75)	3.02 (0.84 1.57‐4.66)

a NYU: New York University.

b REST: rapid eye screening test.

The functionality section had the highest overall rating of the MARS sections with a mean rating of 3.65 (SD 0.75) for Android and 3.71 (SD 0.82) for iOS apps. This indicates that most apps were accurate in performance and user-friendly. Conversely, the subjective quality section had the lowest ratings, with a mean rating of 2.09 (SD 1.01) for Android and 2.21 (SD 1.05) for Apple apps. This suggests the raters perceived many of the apps as low quality and unlikely to be relevant or useful to themselves or others.

The overall mean MARS ratings for Android apps ranged from 1.51 (SD 0.5) to 4.64 (SD 0.39). For Apple apps, ratings ranged from 1.57 (SD 0.46) to 4.66 (SD 0.37). Three apps on the Google Play Store received mean overall MARS ratings above 4.00: Eye Handbook, Eye Patient, and Peek Acuity. Three apps on the Apple App Store were also found to have an overall rating over 4.00: Eye Handbook, Eye Patient, and LooC–Mobile eye test. Two apps on the Google Play Store had overall mean MARS ratings below 2.00: Eye Chart Mobile and Eye Test Exam. Three Apple Store apps were given overall mean MARS ratings below 2.00: EyeTesterFree, eyeTests Easy, and Telehealth Eye Test.

Pearson correlation coefficients between MARS scores, number of reviews, and star ratings are shown in Tables5  6. No significant correlation was found between MARS scores of any section with the number of reviews or star ratings for the Google Play Store VA apps. For the Apple App Store VA apps, however, only the subjective quality MARS scores showed a significant moderate positive correlation with the number of reviews on the app store. All of the MARS sections of the Apple VA apps showed significant moderate or strong correlations between MARS scores and star ratings. The strongest correlation between MARS score and star rating was seen with the aesthetics section (*r*=0.77; *P*<.001).

**Table 5.** Google Play Store: Pearson correlation coefficients between Mobile App Rating Scale (MARS) scores, number of reviews, and star ratings.

MARS	Reviews	Star rating	*P* value^a^	*P* value^b^
Engagement	0.07	0.52	.75	.07
Functionality	0.14	0.53	.52	.07
Aesthetics	0.00	0.39	.00	.23
Information	0.13	0.39	.56	.19
Subjective quality	−0.04	0.45	.86	.13
Total mean score	0.41	−0.35	.05	.24

a Correlation between MARS scores and number of reviews.

b Correlation between MARS scores and star ratings.

**Table 6.** Apple App Store: Pearson correlation coefficients between Mobile App Rating Scale (MARS) scores, number of reviews, and star ratings.

MARS	Number of reviews	Star rating	*P* value^a^	*P* value^b^
Engagement	0.44	0.66	.06	.004
Functionality	0.13	0.64	.60	.005
Aesthetics	0.28	0.77	.24	<.001
Information	0.45	0.76	.06	<.001
Subjective quality	0.49	0.57	.04	.02
Total quality	0.39	0.72	.10	.001

a Correlation between MARS scores and number of reviews.

b Correlation between MARS scores and star ratings.

## Discussion

### Summary of Key Findings

This study aimed to evaluate the quality and features of VA apps available on iOS and Android mobile devices using the MARS. We systematically reviewed apps from both the Google Play Store and Apple App Store, including 23 (52%) Android and 21 (48%) iOS apps. While previous studies have explored the accuracy or limited usability of select VA apps [1,4,53], this is the first to use the validated MARS tool to comprehensively assess both app quality and functionality across a broad sample. This provides a more structured and generalizable assessment of app usability in teleophthalmology.

Our analysis revealed that the most common VA tests were Tumbling E, Snellen Chart, and Landolt C, with other prevalent tests including color vision, astigmatism, Amsler grid, and contrast tests. The mean overall MARS score was 3.04 (SD 0.80) for Android and 3.02 (SD 0.84) for iOS, indicating similar overall quality across both platforms. This score was comparable to those reported in other studies using the MARS, where overall mean scores ranged from 3.03 to 3.67 [7,8,10,11]. App tests and features were also noted to be similar between the platforms. However, there were notable differences between app stores. Many apps on the Apple App Store were older and had not been updated recently, unlike apps on the Google Play Store. In addition, statistically significant correlations between MARS scores, number of reviews, and star ratings were found only for iOS apps. This suggests that reviews should perhaps be given greater consideration on the Apple App Store compared to the Google Play Store when searching for high-quality VA apps.

Although the MARS ratings for general functionality and ease of use of these apps were relatively high, the subjective quality ratings were notably low for both Android and iOS apps. This indicates that while most apps are user-friendly and perform their intended functions accurately, low subjective-quality ratings reveal that users may not find these apps personally valuable or relevant. This discrepancy highlights a gap between technical performance and user satisfaction. The wide range of scores among apps for each MARS section also implies significant heterogeneity in the quality of existing VA apps.

The apps that received overall MARS ratings above 4.0 included Eye Handbook and Eye Patient on both app stores, as well as Peek Acuity on Google Play Store and LooC–mobile eye test on the Apple App Store. Eye Handbook is a provider-targeted app with a multitude of vision tests and educational materials that make it a valuable tool in both clinical and hospital settings [54]. Though not validated in a clinical trial or research study, it is enhanced with many links to journals and ophthalmologic organization websites such as the American Academy of Ophthalmology, American Glaucoma Society, the American Society of Cataract and Refractive Surgery, and the Association for Research in Vision and Ophthalmology. Eye Patient, developed by the same publisher as Eye Handbook, is a patient-focused vision testing app. It similarly includes a variety of tests with ample educational materials. It also features a directory of eye care providers and options to keep track of appointments, medications, and refractions.

Peek Acuity is a clinically validated app designed for testing distance vision [4,55-60]. The app’s sophisticated design, clear instructions, and customization options for letter size, test distance, and VA units make it a reliable tool for professional use in clinical settings. However, its use requires an operator to hold the device away from the patient, precluding patient self-administration. In addition, the app lacks other vision tests and features, limiting its comprehensive utility.

LooC–Mobile Eye Test received the highest overall MARS rating, likely due to its sleek design and intuitive features suitable for a wide audience. The app supports both near and distance VA testing by connecting to a tablet or computer browser. Notably, it uses the smartphone’s facial recognition technology to ensure accurate distance measurement for both near and distance VA tests. The app also includes color vision testing and an Amsler grid, although it lacks the extensive range of features offered by other apps such as the Eye Handbook. While its current features suggest potential suitability for clinical VA testing, clinical validation is necessary to ensure its reliability compared to traditional methods. A previous study reported that the app has been implemented in German clinics, demonstrating favorable results anecdotally [4].

The findings of this study have significant implications for the use of VA apps in clinical practice and teleophthalmology. The high functionality scores indicate that many VA apps are technically sound and easy to use, making them suitable for quick VA assessments by both ophthalmologists and non-ophthalmologist providers. Though the majority of apps were targeted towards providers, many may also be used by patients for monitoring changes in VA. These apps can be valuable tools, particularly in settings with limited access to eye care providers.

One notable concern, however, is the lack of robust clinical validation for most VA apps [53]. Out of 44 apps reviewed, only 5 (11%) had clear evidence of clinical validation. While previous studies have shown that VA app test results are comparable to standard VA tests [1,55,61-63], clinical validation may also be useful in determining app inferiority in some cases. For example, Satgunam et al [60] showed the SmartOptometry VA app was less accurate than standardized VA testing or the Peek Visual Acuity app. However, clinical validation may be challenging for many freely available VA apps. Several apps evaluated in this study used unconventional testing methods, such as sentence or number recognition or multiple-choice tests. Test results were often nonstandardized as well, with scores appearing as percentages or simplistic ratings such as “good.” Such results raise questions about their reliability and effectiveness in a clinical or home setting. This underscores the need for rigorous clinical testing and validation of VA apps to ensure they meet the necessary standards for provider use and recommendation to patients.

### Recommendations for Future Research and App Development

In addition to clinical validation, this study highlights several key components of high-quality apps that should be prioritized in future development. While many apps incorporate basic tests like VA and color vision, few are equipped for comprehensive eye examinations. An ideal app for health care providers would integrate all the features identified in this study, including less common ones such as pupillary distance measurement, visual fields, and stereopsis tests. Clear instructions for each test are crucial for accurate results. Patient-oriented apps should provide straightforward instructions and a user-friendly design accessible to individuals of all ages and technological abilities [64]. Educational materials, though often underused, can significantly enhance patient understanding of eye diseases. In addition, offering multiple language options would greatly increase the app’s accessibility and effectiveness for patients from diverse nationalities and ethnic backgrounds.

Some apps incorporated the smartphone’s camera and facial recognition capabilities to ensure the VA tests were taken at an appropriate distance from the screen. Taking advantage of these developing technologies can increase test accuracy and reliability. As artificial intelligence continues to develop, its integration into VA apps will likely further improve their effectiveness as tools in teleophthalmology [65,66].

### Limitations

Several limitations must be considered in this study. The exclusion of paid apps and apps that require a secondary device means that other high-quality and clinically relevant VA apps may have been overlooked. The exclusion of non-English apps may also have resulted in the omission of potentially high-quality apps used by non-English speaking populations. These limitations may reduce the comprehensiveness of our review, but the exclusion criteria were chosen to include apps that were most generalizable to the majority of patients and providers. Another limitation is the potential for bias in the app selection process, as it relied on the availability and discoverability of apps on the app stores at the time of the search. Nonetheless, both app stores were thoroughly searched, and the relatively large sample size of 44 total apps increases the generalizability of our findings. Furthermore, our analysis was limited to publicly available information from the app stores and the apps themselves, without deeper insights into the development processes or clinical testing that may have been conducted. It should also be noted that using separate raters for Android and Apple apps might limit direct comparisons between app stores. However, the increased number of raters further validates the overall results of the study, especially since several included apps were present on both app stores.

### Conclusions

This study offers valuable insights for providers and patients seeking to use vision testing apps. This is the first study to use the MARS to rate the quality of freely available VA apps. The results of this study revealed significant variability in the quality and features of VA apps available on both the Google Play Store and Apple App Store. The overall quality of these apps is comparable across platforms, yet the necessity for clinical validation to ensure reliability and accuracy remains evident. Functionality scores were the highest among MARS sections, but subjective quality was low, most likely due to the limited clinical relevance of most apps. Future app development should focus on integrating comprehensive eye examination features with clear instructions, educational materials, and accessibility in multiple languages. Such improvements could transform VA apps into more effective tools for teleophthalmology and patient self-monitoring, thereby improving access to visual health care on a global scale.

## Supplementary material

10.2196/65997 Multimedia Appendix 1 Extracted data and Mobile App Rating Scale (MARS) scores.

10.2196/65997 Checklist 1 PRISMA checklist.

## Abbreviations

MARS: Mobile App Rating Scale
PRISMA: Preferred Reporting Items for Systematic Reviews and Meta-Analyses
VA: visual acuity
